# Underlying pharmacological mechanisms of psilocin-induced broadband desynchronization and disconnection of EEG in rats

**DOI:** 10.3389/fnins.2023.1152578

**Published:** 2023-06-22

**Authors:** Filip Tylš, Čestmír Vejmola, Vlastimil Koudelka, Václava Piorecká, Lukáš Kadeřábek, Marcel Bochin, Tomáš Novák, Martin Kuchař, Zdeňka Bendová, Martin Brunovský, Jiří Horáček, Tomáš Pálení ček

**Affiliations:** ^1^Psychedelic Research Centre, National Institute of Mental Health, Klecany, Czechia; ^2^3rd Faculty of Medicine, Charles University in Prague, Prague, Czechia; ^3^Faculty of Biomedical Engineering, Czech Technical University in Prague, Kladno, Czechia; ^4^Forensic Laboratory of Biologically Active Substances, Department of Chemistry of Natural Compounds, University of Chemistry and Technology Prague, Prague, Czechia

**Keywords:** serotonergic psychedelics, psilocybin/psilocin, model of acute psychosis, quantitative EEG, power spectra, phase-lagged coherence, global functional connectivity (GFC)

## Abstract

**Introduction:**

Psilocybin is one of the most extensively studied psychedelic drugs with a broad therapeutic potential. Despite the fact that its psychoactivity is mainly attributed to the agonism at 5-HT_2A_ receptors, it has high binding affinity also to 5-HT_2C_ and 5-HT_1A_ receptors and indirectly modulates the dopaminergic system. Psilocybin and its active metabolite psilocin, as well as other serotonergic psychedelics, induce broadband desynchronization and disconnection in EEG in humans as well as in animals. The contribution of serotonergic and dopaminergic mechanisms underlying these changes is not clear. The present study thus aims to elucidate the pharmacological mechanisms underlying psilocin-induced broadband desynchronization and disconnection in an animal model.

**Methods:**

Selective antagonists of serotonin receptors (5-HT_1A_ WAY100635, 5-HT_2A_ MDL100907, 5-HT_2C_ SB242084) and antipsychotics haloperidol, a D_2_ antagonist, and clozapine, a mixed D_2_ and 5-HT receptor antagonist, were used in order to clarify the underlying pharmacology.

**Results:**

Psilocin-induced broadband decrease in the mean absolute EEG power was normalized by all antagonists and antipsychotics used within the frequency range 1–25 Hz; however, decreases in 25–40 Hz were influenced only by clozapine. Psilocin-induced decrease in global functional connectivity and, specifically, fronto-temporal disconnection were reversed by the 5-HT_2A_ antagonist while other drugs had no effect.

**Discussion:**

These findings suggest the involvement of all three serotonergic receptors studied as well as the role of dopaminergic mechanisms in power spectra/current density with only the 5-HT_2A_ receptor being effective in both studied metrics. This opens an important discussion on the role of other than 5-HT_2A_-dependent mechanisms underlying the neurobiology of psychedelics.

## 1. Introduction

Psilocybin is a naturally occurring psychedelic/hallucinogen that has been recently given a lot of attention especially as a potential therapeutic drug for the treatment of several neuropsychiatric disorders including depression (Tylš, 2015; Carhart-Harris et al., 2016a,^2021^; Mahapatra and Gupta, 2017). The psychedelic effects of psilocybin in humans can be characterized by changes in perception, thought, emotions and cognitive processes (Tyls et al., 2014). It has been shown that several phenomenological signs/symptoms of intoxication have correlates in brain activity (Carhart-Harris and Friston, 2010; Nour et al., 2016). This study focuses on a highly translatable measure of brain activity across species – quantitative EEG (qEEG) (Forrest et al., 2014) in order to deepen the understanding of the mechanisms of psilocybin action. Psilocybin and its active metabolite psilocin is a tryptamine psychedelic with predominant activity on 5-HT_2A/C_ and 5-HT_1A_ receptors and with minimal effect on other neurotransmitter systems (Tyls et al., 2014; Nichols, 2016). 5-HT_2A_ receptor is definitely the most important receptor underlying the psychedelic effects of serotonergic psychedelics in humans and the behavioral effects in rodents (Glennon et al., 1984; Vollenweider et al., 1998b; Madsen et al., 2019). Despite this fact, psilocybin can also secondarily induce an activation of the dopaminergic system (Vollenweider et al., 1998b).

Already the very early visually evaluated EEG studies with serotonergic psychedelics described a desynchronization of EEG represented by decreased amplitude and increased frequency of resting-state EEG in animals (Bradley and Elkes, 1957; Vogt et al., 1957; Speck, 1958; Meldrum and Naquet, 1971) as well as humans (Chweitzer et al., 1936; Gastaut et al., 1953; Goldstein et al., 1963; Shagass, 1966; Fink, 1969) [reviewed in Tyls et al., 2014]. This refers to a desynchronization of resting-state alpha activity similar to what happens with the eye-opening reaction. Congruently, recent human studies using qEEG approaches looking at the effect of psilocybin, LSD (*N,N*-diethyl-D-lysergamide), DMT (*N,N*-dimethyltryptamine) and ayahuasca (traditional psychedelic brew containing DMT and MAO-A inhibitors) found decreases in theta (Don et al., 1998; Riba et al., 2002; Timmermann et al., 2019) and alpha oscillations (Riba et al., 2002; Kometer et al., 2013; Muthukumaraswamy et al., 2013; Schenberg et al., 2015; Carhart-Harris et al., 2016b; Timmermann et al., 2019; Pallavicini et al., 2020). Some also documented an increase in high-frequency activity (Don et al., 1998; Schenberg et al., 2015; Pallavicini et al., 2020); however, these effects are still disputable as they can be partially explained by increased muscular tension. More recent animal studies were conducted using another potent psychedelic – 5-HT_2A/C_ agonist DOI (2,5-dimethoxy-4-iodoamphetamine), which decreased delta and gamma activity (Celada et al., 2008; Wood et al., 2012). Furthermore, a lot of new findings in humans congruently describe that psychedelics induce unpredictable neuronal activity, disorganization of neuronal networks and a variety of changes in functional connectivity (Carhart-Harris et al., 2012, 2016b; Muthukumaraswamy et al., 2013; Petri et al., 2014; Tagliazucchi et al., 2014; Schartner et al., 2017; Preller et al., 2019, 2020). Our recent qEEG study (Vejmola et al., 2021) found an identical pattern of changes (broadband decrement in EEG absolute power and disconnection in the frequency range 0.5–40 Hz) following treatment with phenethylamine as well as tryptamine psychedelics [psilocin, LSD, 2,5-dimethoxy-4-bromoamphetamine (DOB) and mescaline] and we have also previously seen comparable effects following treatment with 4-bromo-2,5-dimethoxyphenethylamine (2C-B) (Palenicek et al., 2013; Vejmola et al., 2021).

Despite the known receptor profile of psilocybin/psilocin, the receptor mechanisms underlying these qEEG changes are not clear. It is worth mentioning that in our previous study we found an important contribution not only of 5-HT_2A_ but also of 5-HT_1A_ and 5-HT_2C_ receptors in the behavioral effects of psilocin (Tyls et al., 2016). Therefore, the main aim of the present paper is to study the contribution of these serotonin receptors to psilocin’s effect on brain EEG activity and connectivity. In order to reveal these mechanisms, we modulated the effects of psilocin on qEEG with selective serotonin antagonists (5-HT_2A_, 5-HT_1A_, 5-HT_C_) and with commonly used antipsychotic drugs (D_2_ antagonist haloperidol and multi-receptor antagonist clozapine). We hypothesize that psilocin-induced changes will be driven mainly by 5-HT_2A_ receptors while the contribution of other receptor mechanisms cannot be ruled out. The investigation of the receptor mechanisms underlying the effect of psilocin on brain function is key to the evaluation of its antidepressant properties and, by extension, to the potential introduction of psilocybin into clinical practice.

## 2. Materials and methods

### 2.1. Animals

The experiments were performed on adult male Wistar rats (SPF Velaz, s.r.o., Czechia) weighing 280–300 g at the time of surgery. The size of the groups was chosen upon the previous experience, taking into account the 3Rs principles. Rats were randomly assigned to experimental groups. The acclimatization period in an in-house animal facility lasted 7–10 days prior to surgery. The animals were housed in pairs and were regularly handled every second day. The recovery from surgery lasted 7 days. The rats were then housed individually in cages in order to prevent damage to implanted electrodes and weight gain was monitored as a marker of good recovery. Constant housing conditions were guaranteed during the entire period: 12-h light/dark regime, temperature 22 ± 2°C, humidity 40 ± 10%, free access to the standard diet and water. All experiments respected the Guidelines of the European Union (86/609/EU) and followed the instructions of the National Committee for the Care and Use of Laboratory Animals.

### 2.2. Drugs

Psilocin (PSI, THC-Pharm GmbH, Germany) was dissolved in 2 ml of saline (0.9% NaCl) acidified by 10 μl of glacial acetic acid, subsequently adjusted to a volume of 5 ml with saline and administrated in a dose of 4 mg/kg subcutaneously (SC). MDL100907 tartrate 0.5 mg/kg SC (a2A, ABX, GmbH Germany) was dissolved in saline, SB 242084 dihydrochloride 1 mg/kg SC (a2C, Tocris, UK) in cyclodextrin (1 g dextrin + 52 mg citric acid + 10 ml saline, buffered by NaOH to pH 7.4) and WAY100635 maleate 1 mg/kg SC (a1A, Sigma-Aldrich, Germany). Haloperidol 0.1 mg/kg SC (HAL, Sigma-Aldrich, Germany) was dissolved in isotonic glucose solution, and intraperitoneal (IP) clozapine 5 mg/kg, (CLO, Sigma-Aldrich, Germany) was dissolved in saline acidified by a small amount of 0.1 M HCl. Saline served as a vehicle. All drugs were administered in a volume of 2 ml/kg of animal weight. The investigator was not blinded to the treatment.

### 2.3. Stereotactic surgery

**FIGURE 1:**
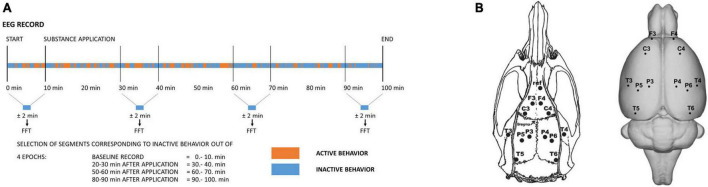
**(A)** Schematic drawing illustrating the time course of the experiment and the selection of signals for further analysis. Four epochs were selected. Out of each 10-min epoch, only artifact-free EEG signals corresponding to behavioral inactivity segments altogether lasting ± 2 min were selected and further subjected to Fast Fourier Transform and subsequent analysis. **(B)** Layout and labeling of the electrodes (el.) on the rat’s skull (left) and brain (right). Coordinates: A +5 mm and L ±2 mm for the frontal association cortex (el. F3/F4), A +2.2 mm and L ±3.2 mm for the primary motor cortex (el. C3/C4), A –3.8 mm and L ±2.5 mm (el. P3/P4) for the medial parietal association cortex, A –4.5 mm and L ±4.5 mm (el. P5/P6) for the lateral parietal association cortex, A –3.6 mm and L ±7.2 mm for the secondary auditory cortex (el. T3/T4), and A –8.3 mm and L ± 5.8 mm for the temporal association cortex (el. T5/T6).

The methodology was already described in detail in our previous studies (Palenicek et al., 2011, 2013; Vejmola et al., 2021). In brief, rats under isoflurane anesthesia were stereotactically implanted with 14 gold-plated electrodes. Twelve electrodes were placed on homologous areas on the surface of the frontal, parietal and temporal cortex of the left and right hemispheres established based on the stereotactic atlas (positions shown in Figure 1A) (Paxinos and Watson, 2007). The reference electrode was implanted above the olfactory bulb and the ground electrode subcutaneously in the occipital region. Electrodes were fixed to the rats’ skulls with dental cement (Dentalon). Connectors enabling linkage to the registration system were connected to the electrodes under short-term isoflurane anesthesia 1 day before registration.

### 2.4. EEG recording

Of the 12 animals operated on for each group, at least 8 animals were subsequently used. The exclusion criteria were insufficient weight gain, marks of postoperative inflammation or insufficient EEG signal transmission (e.g., continual electrode artifacts in any channel). Each rat was recorded only once with a specific treatment. The experiments were conducted during the daytime between 8:00 and 14:00. The animals were connected to the registration system in an electromagnetically shielded experimental room in their home cages. They were able to move freely during the whole recording session. After an initial 10 min of baseline recording, the compounds were administered in the following order: vehicle, psilocin alone or psilocin + serotonin antagonist/antipsychotic. Recording continued for another 90 min. EEG signal was recorded with a 250 Hz sampling rate using BrainScope EADS-221 amplifier and data acquisition system (Unimedis, Prague, Czechia). The signal was hardware bandpass-filtered within the range of 0.15–70 Hz, and the acquired data were of 16-bit depth, 7.63 nV/bit resolution (i.e., ∼130 bit/μV) and a dynamic range of ± 500 μV. Episodes of behavioral activity and inactivity were co-registered during the acquisition of EEG and the epochs in the trace during which the animals were handled (administration of substances, manipulation with electrodes) and during which sleep was suspected were marked as artifacts and excluded from the analysis (Figure 1).

### 2.5. EEG signal preprocessing and analysis

The data was further digitally filtered by a finite impulse response bandpass filter with 111 coefficients in the 0.5–40.0 Hz range and pre-processed in WaveFinder v.2.6 (Unimedis, Prague) (Krajèa et al., 1993). In order to describe the dynamics of EEG changes, 4 epochs of the EEG signal were used: 10 min of baseline recording and 3 epochs after treatments (20–30 min, 50–60 min, 80–90 min). The EEG signal was divided into parts corresponding to behavioral activity and inactivity and, in order to work with the data model “resting-state EEG” (Fujakova et al., 2014; Vejmola et al., 2021), only signals corresponding to behavioral inactivity were subjected to further analyses in Neuroguide Deluxe v.2.6.5 software (Applied Neuroscience Inc.). Initially, characteristic artifact-free samples of a total length of 10 s of the EEG signal were visually selected from each of the time epochs so that they included all specific patterns of EEG within the recorded signal. Subsequently, a semiautomatic selection using a specific Neuroguide built-in tool was performed to select ∼2 min of artifact-free signal. The semiautomatic selection was based on the amplitude (criteria: 1.25 of maximal amplitude; split-half reliability ≥0.9; test-retest reliability ≥0.9) and was followed by visual inspection in order to exclude potential artifacts. The minimum length of selected signal accepted for analyses was 30 s. After fast Fourier transformation (FFT), EEG power and coherence (phase-lagged) analyses were performed for each Hz and in the following frequency bands: delta 1–4 Hz, theta 4–8 Hz, alpha 8–12 Hz, beta 12–25 Hz, high beta 25–30 Hz, gamma 30–40 Hz.

### 2.6. Statistics and visualization of behavioral activity

Behavioral activity was assessed as the ratio of active behavior to inactivity in each 10-min epoch. The time intervals between the markers’ positions were measured and summed up in every individual interval for each treatment and also baseline. We compared the length of inactive episodes between baseline and related treatment intervals. Shapiro test was used for heterogeneity and QQ plot for normality of the preprocessed dataset. Data had normal distribution, therefore we used analysis of variance for repeated measures (RM-ANOVA) followed by Bonferroni *post-hoc* test for each treatment condition. Pie charts depicting the behavioral activity of the rats are a plot of the average ratio (group per time epoch) of time spent actively/inactively as scored during the EEG recording.

### 2.7. Statistics and visualization of qEEG

Due to high interindividual variability, the computed data were normalized by the Box-Cox Ratio transformation with λ = 0 (John and Draper, 1980; Koopman et al., 1996) and then tested by Shapiro-Wilk’s test to ensure normality. In order to decrease type II error–and because we were not interested in how the various antagonists interacted with each other–each combination of psilocin with antagonist/antipsychotic was analyzed separately. Thus, five runs (a1A/a2A/a2C/HAL/CLO) of analyses were performed in total (“antagonist/antipsychotic group”). Each frequency band was assessed separately. RM-ANOVA with the Greenhouse-Geisser correction was used in order to compare the effect of specific treatments (between-subject factor) and in time (within-subject factor) on the mean (averaged over all electrodes) spectral power, pairwise coherences, and global functional connectivity (GFC). The significance level was set to *p* < 0.05. *Post-hoc* tests corrected for multiple comparisons by the Bonferroni method were then applied in cases of statistically significant interactions between factors “treatment” and “time.” In order to assess the global effect of psilocin and its combination with antagonist/antipsychotic treatments on GFC, *post-hoc* tests were applied and interpreted to determine if there was a significant main effect of factor “treatment” and no significant interaction between factors “treatment” and “time” in the RM-ANOVA model. The Matlab built-in statistical toolbox was used for all statistical analyses.

Mean values of absolute EEG power spectra difference were plotted in graphs using the GraphPad Prism 8 software. Topographic maps depicting the distribution of significant spectral power change were created using the method of 3D spline mapping (Piorecka et al., 2018). The scripts used to perform the spline mapping are available on GitHub (DOI: 10.5281/zenodo.4059491)^1^ and archived in Zenodo. Statistical evaluation was performed separately for each point of the map.

The Wistar rat brain model was used for brain mapping purposes: see Brain Atlas Reconstructor (BAR)^2^ (Majka et al., 2013). Coherence visualizations were performed in the Python software. Figures 4, 5 depict the raw effect by the line width, with the colors representing the differences between the group means of coherences after the Box-Cox correction with λ = 0. The higher the group mean of the Box-Cox-corrected coherences, the higher the mean relative difference from the baseline in a group. The scale for depicting coherence was set with respect to the maximal and minimal differences across all conditions. We characterized a qualitative GFC pattern by plotting a probability distribution of all relative connectivity changes from the baseline. Each distribution was obtained by the Kernel Density Estimator (KDE) applied to concatenated coherence values across all subjects, electrode pairs, and frequency bands. The Gaussian kernel of 0.5 width was used in this case. Quantitative change in GFC was measured by averaged lagged coherence changes across electrode pairs and frequency bands separately within each subject.

## 3. Results

**FIGURE 2:**
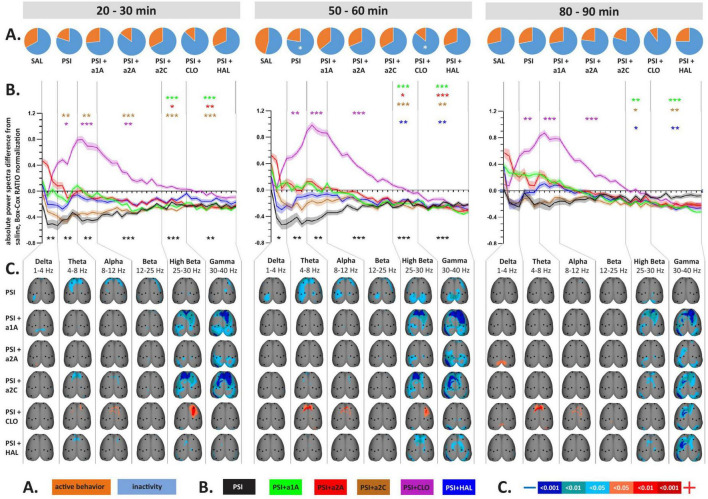
Impact of 5-HT antagonists and antipsychotics on EEG absolute power changes induced by psilocin. The effects of treatments in three separate time epochs, as indicated. The abbreviations represent the following: SAL (control group, saline 2 ml/kg), PSI (psilocin, 100 mg/kg), a1A (5-HT_1A_ antagonist, 1 mg/kg), a2A (5-HT_2A_ antagonist, 0.5 mg/kg), a2C (5-HT_2C_ antagonist, 1 mg/kg), CLO (clozapine 5 mg/kg), HAL (haloperidol 0.1 mg/kg). The level of significance is indicated as *p* < 0.05 = *. **(A)** Pie charts depicting behavioral activity–average time the rats spent active (orange) and inactive (blue); **(B)** Mean values of absolute EEG power spectra difference against the control group normalized by the Box-Cox method for all substances over consecutive time epochs; the x-axis shows the frequency in Hz, the y-axis the dimensionless quantity. The level of significance is indicated as *p* < 0.05 = *, *p* < 0.01 = **, *p* < 0.001 = ***. Lines show the mean values, shaded error bars correspond to standard error mean values. **(C)** EEG topographic maps of absolute power spectra differences–all treatments vs. the control group, drugs in rows, frequency bands in columns; only significant changes are indicated. The direction of change is indicated by colors–decreases in blue, increases in red. The level of significance is indicated by a three-level-scaled color spectrum (see legend). Corresponding legends **(A–C)** are at the bottom of the figure. Data were corrected for multiple comparisons via Bonferroni *post-hoc* test in case of time and treatment correction.

**FIGURE 3:**
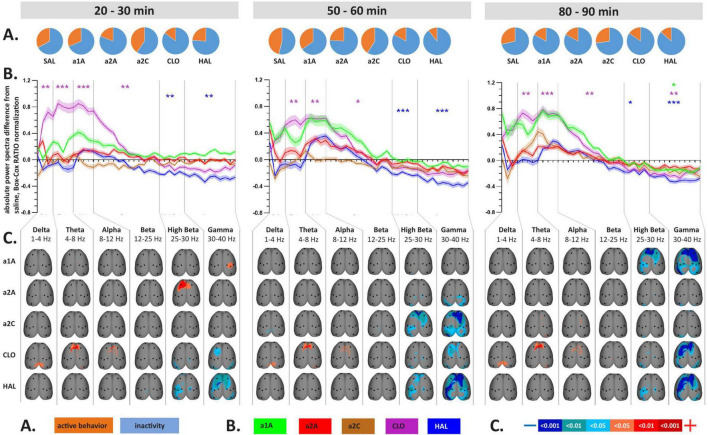
Impact of 5-HT antagonists and antipsychotics on EEG absolute power changes. The effects of treatments in three separate time epochs, as indicated. The abbreviations represent the following: SAL (control group, saline 2 ml/kg), PSI (psilocin, 100 mg/kg), a1A (5-HT_1A_ antagonist, 1 mg/kg), a2A (5-HT_2A_ antagonist, 0.5 mg/kg), a2C (5-HT_2C_ antagonist, 1 mg/kg), CLO (clozapine 5 mg/kg), HAL (haloperidol 0.1 mg/kg). Panels **(A–C)** are described in Figure 2. Data were corrected for multiple comparisons via Bonferroni *post-hoc* test in case of time and treatment correction.

**FIGURE 4:**
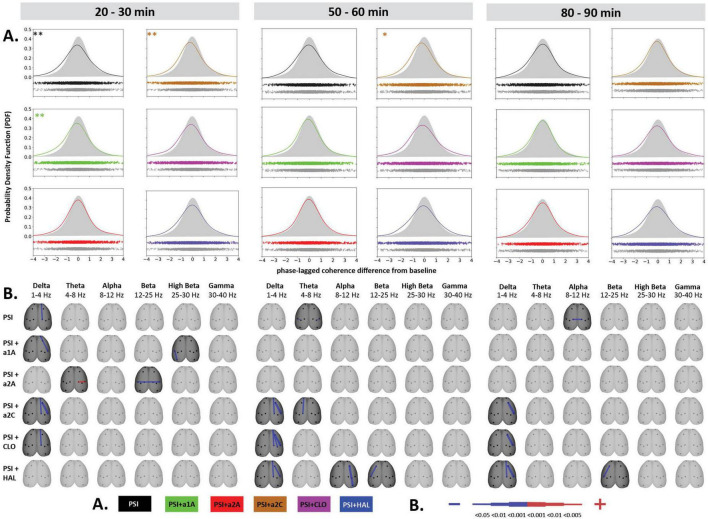
Impact of 5-HT antagonists and antipsychotics on EEG phase-lagged coherence changes induced by psilocin. The effects of treatments in three separate time epochs, as indicated. The abbreviations represent the following: SAL (control group, saline 2 ml/kg), PSI (psilocin, 100 mg/kg), a1A (5-HT_1A_ antagonist, 1 mg/kg), a2A (5-HT_2A_ antagonist, 0.5 mg/kg), a2C (5-HT_2C_ antagonist, 1 mg/kg), CLO (clozapine 5 mg/kg), HAL (haloperidol 0.1 mg/kg). **(A)** Global phase-lagged coherence connectivity. Each figure contains estimated probability distributions of connectivity changes in the drug (solid line) and control (filled area) conditions. A negative or positive change of GFC in a drug condition is indicated by its shifted distribution toward negative or positive values with respect to the control condition. The scatterplots below each graph represent functional connectivity data across subjects, electrode pairs and frequency bands used by the KDE. All target groups distributions are shown in color and are compared to the control group (saline 2 ml/kg) shown in gray in each graph. The level of significance of between-group differences is indicated as *p* < 0.05 = *, *p* < 0.01 = ^**^. **(B)** EEG phase-lagged coherence topographic maps. All treatments are compared against the control group, drugs in rows, frequency bands in columns. Only significant changes are indicated (*p* < 0.05). The direction of change is indicated by colors–decreases in blue, increases in red. The level of relative difference is indicated by a three-level-scaled line width. Corresponding legends **(A,B)** are at the bottom of the figure. Data were corrected for multiple comparisons via Bonferroni *post-hoc* test in case of time and treatment correction.

**FIGURE 5:**
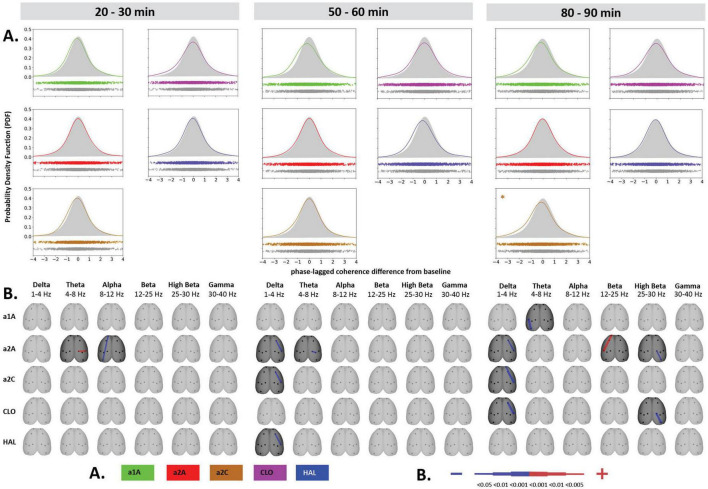
Impact of 5-HT antagonists and antipsychotics on EEG phase-lagged coherence changes. The effects of treatments in three separate time epochs, as indicated. The abbreviations represent the following: SAL (control group, saline 2 ml/kg), PSI (psilocin, 100 mg/kg), a1A (5-HT_1A_ antagonist, 1 mg/kg), a2A (5-HT_2A_ antagonist, 0.5 mg/kg), a2C (5-HT_2C_ antagonist, 1 mg/kg), CLO (clozapine 5 mg/kg), HAL (haloperidol 0.1 mg/kg). The level of significance of between-group differences is indicated as *p* < 0.05 = *. Panels **(A,B)** are described in Figure 4. Corresponding legends **(A,B)** are at the bottom of the figure. Data were corrected for multiple comparisons via Bonferroni *post-hoc* test in case of time and treatment correction.

The effects of psilocin treatment alone have been already included in our previous manuscript comparing the EEG effects of tryptamine and phenethylamine psychedelics (Vejmola et al., 2021). EEG data of individual groups of substances are shown in comparison to the control group (Figures 2–5), relative changes compared to psilocin can be found in the Supplementary Figure 1.

### 3.1. Behavioral activity

Analysis of variance for repeated measures showed a significant effect in the CLO group only, where we found the effect of treatment *F*(3,7) = 18.8, *p* < 0.001, and time *F*(3,21) = 3.24, *p* < 0.05, but no interaction between factors. *Post-hoc* analyses showed that psilocin-induced decreases in behavioral activity were to some extent also present after co-administration with CLO during the second temporal window (Figures 2A, 3A).

### 3.2. EEG absolute mean power

Analysis of variance for repeated measures on power spectra revealed a significant interaction of all treatments:time for majority of frequency bands, specifically, see Table 1. PSI-induced mean power decreases across frequencies, which were significant during the first two temporal windows, were not fully restored by any of the drugs used. Within the range of 1–25 Hz, a1A, a2A, and HAL restored psilocin-induced power decreases in both temporal windows while a2C was effective only at 50–60 min. At the same time, none of the antagonists altered the power decreases within the frequency bands 25–40 Hz except HAL (only at 20–30 min) and CLO. It is of note that except HAL, which alone decreased the power in 25–40 Hz, none of the other antagonists alone induced changes in mean power within this frequency range during the first and second temporal windows. CLO alone, as a multi-receptor agent, had a very specific effect characterized by a global power increase in the 1–25 Hz range. This effect was dominant also in co-administration with psilocin, technically fully reversing/masking its effects in the 4–25 Hz range (theta, alpha and beta bands). Interestingly, a similar shape of power increases, though not statistically significant, was produced by a1A. For exact *p*-values see Figures 2B, 3B.

**TABLE 1:** Results of RM-ANOVA on power spectra.

RM ANOVA	Band	Frequency range	df (treatment:time)	df (error time)	*F*	*p*-value
**a1A**	Delta	1–4 Hz	6	60	1,79	n.s.
	Theta	4–8 Hz	6	60	2,10	n.s.
	Alpha	8–12 Hz	6	60	1,43	n.s.
	Beta	12–25 Hz	6	60	1,79	n.s.
	**High beta**	25–30 Hz	6	60	5,91	***
	**Gamma**	30–40 Hz	6	60	7,93	***
**a2A**	Delta	1–4 Hz	6	62	2,08	n.s.
	Theta	4–8 Hz	6	62	2,22	n.s.
	**Alpha**	8–12 Hz	6	62	2,29	*
	**Beta**	12–25 Hz	6	62	3,58	**
	**High beta**	25–30 Hz	6	62	2,69	*
	**Gamma**	30–40 Hz	6	62	2,68	*
**a2C**	Delta	1–4 Hz	6	64	2,21	n.s.
	**Theta**	4–8 Hz	6	64	3,21	**
	**Alpha**	8–12 Hz	6	64	3,20	**
	**Beta**	12–25 Hz	6	64	3,21	**
	**High beta**	25–30 Hz	6	64	2,37	*
	**Gamma**	30–40 Hz	6	64	4,59	***
**CLO**	**Delta**	1–4 Hz	6	62	4,29	**
	**Theta**	4–8 Hz	6	62	3,68	**
	**Alpha**	8–12 Hz	6	62	3,49	**
	**Beta**	12–25 Hz	6	62	4,78	***
	**High beta**	25–30 Hz	6	62	3,57	**
	**Gamma**	30–40 Hz	6	62	5,21	***
**HAL**	Delta	1–4 Hz	6	66	2,01	n.s.
	**Theta**	4–8 Hz	6	66	2,53	*
	**Alpha**	8–12 Hz	6	66	2,75	*
	**Beta**	12–25 Hz	6	66	3,70	**
	**High beta**	25–30 Hz	6	66	2,83	*
	**Gamma**	30–40 Hz	6	66	4,44	***

The table shows the results of RM-ANOVAs for 3 antagonist groups (a1A, a2A, a2C) and 2 antipsychotic groups (CLO, HAL). Significant interactions are marked in bold. The level of significance is indicated as follows: *p* < 0.05 = *, *p* < 0.01 = **, *p* < 0.001 = ***.

### 3.3. Topographic maps of EEG power spectra

The topographic maps show results according to the overall changes in mean power, with the psilocin-induced current density decreases being topographically represented as: (1) bilateral fronto-temporal strips across 1–40 Hz during the first two temporal windows, and (2) parieto-temporal blobs mostly pronounced in the 25–40 Hz range during the second temporal window. The fronto-temporal changes within 1–25 Hz were normalized by all antagonists (a1A, a2A, a2C) as well as CLO. Both antipsychotics (CLO, HAL) were the only two that influenced the changes in 25–40 Hz during the first temporal window and CLO alone also in the second temporal window. In contrast to that, a1A and a2C potentiated the effects within this frequency range resulting in a large current density decreases over large fronto-parieto-temporal areas. CLO further changed the fronto-parietal activity within the 4–30 Hz range resulting in increased current density (Figure 2C). Most of the antagonists alone induced current density decreases observed in the 25–40 Hz range within at least one of the time windows. CLO induced current density increases in 1–12 Hz mainly above the fronto-parietal regions in all time windows. Interestingly, a2A also induced an increase in frontal activity in 25–30 Hz and a1A in 30–40 Hz above the right parietal cortex, both in the first time window (Figure 3C).

### 3.4. EEG lagged-coherences

Psilocin induced shift to the left toward negative GFC values. Regarding the quantitative assessment, RM-ANOVA showed the effect of PSI treatment *F*(1,14) = 9.17, *p* < 0.01, which was significant in the first time window (*p* < 0.01) and almost reached the level of significance in the second time window (*p* = 0.09). The RM-ANOVA showed the effect of the treatment for all antagonist groups [a1A group: *F*(3,28) = 4.33, *p* < 0.05, a2A group: *F*(3,28) = 3.92, *p* < 0.05, a2C group: *F*(3,28) = 6.01, *p* < 0.01]; however, no effect was found for antipsychotics (CLO and HAL). Compared to saline, *post-hoc* tests showed that decreased GFC after psilocin remained significant in PSI + a1A in the first time window (*p* < 0.01) and PSI + a2C in the first (*p* < 0.01) and second (*p* < 0.05) time windows but not in PSI + a2A. The shift to the left toward negative values following PSI treatment was normalized only by a2A, while other drugs had minimal impact. *Post-hoc* tests did not reveal any other changes in GFC including the antagonists/antipsychotics-alone treatments (Figures 4A, 5A). The major isolated decreases in connectivity induced by PSI were present/potentiated with all the 5-HT antagonists as well as CLO and HAL. PSI-induced fronto-temporal disconnection was reversed only by a2A (Figures 4B, 5B).

## 4. Discussion

### 4.1. Main findings

Our experiments showed that all serotonin receptor antagonists (a1A, a2A, a2C), as well as CLO and HAL, when co-administered with PSI, partially normalized psilocin-induced power decrease within low (1–8 Hz) and mid (8–25 Hz) frequencies while only CLO was able to influence power decreases at higher frequencies (25–40 Hz). The changes in global connectivity induced by PSI were reversed only by a2A. Discrete connectivity decreases were effectively attenuated by a2A and partly also a1A.

### 4.2. Putative role of 5-HT_2A_ 5-HT_1A_, and 5-HT_2C_ receptors in the EEG changes induced by psilocin

Human studies have shown that 5-HT_2A_ antagonists block most of the subjective and behavioral effects of psilocybin (Vollenweider et al., 1998a; Carter et al., 2005; Wittmann et al., 2007). Indeed, in rats, 5-HT_2A_ antagonists block the effects of psilocin on the head twitch response (González-Maeso et al., 2007), drug discrimination (Winter et al., 2007), exploratory and investigative behavior (Wing et al., 1990), and sensorimotor gating (Halberstadt et al., 2011). Thus, it appears that the 5-HT_2A_ receptor has a central role in psychedelic-induced changes, whereas the 5-HT_1A_ and 5-HT_2C_ receptors are less important (Halberstadt and Geyer, 2012; Tyls et al., 2016). 5-HT_2A_ receptors are generally located post-synaptically on GABAergic interneurons and glutamatergic pyramidal neurons, primarily in cortical layers III and V (Jakab and Goldman-Rakic, 1998; Hoyer et al., 2002; Santana et al., 2004; Watakabe et al., 2009). Their organization leads to an altered excitatory-inhibitory balance (Moreau et al., 2010), resulting in an overall desynchronization of neuronal firing and, thus, presumably, a reduction in power spectra. Our results confirmed the crucial role of 5-HT_2A_ receptors: psilocin-induced reductions in spectral power up to 25 Hz and brain connectivity were entirely blocked by a2A. These observations are in line with both animal and human studies: a2A completely reversed DOI-induced reductions in low-frequency oscillations (up to 4 Hz) in the PFC in rats (Celada et al., 2008), and pretreatment with another 5-HT_2A_ receptor antagonist ketanserin strongly attenuated psilocybin-induced EEG changes in humans (Kometer et al., 2013). When given alone, a2A tended to increase power spectra up to 20 Hz. This finding is consistent with the literature (Sebban et al., 2002). Furthermore, fronto-temporal connectivity decrease was not reversed by any of the drugs except a2A. Since disconnection is a typical finding in acute psychosis (Friston and Frith, 1995) and fronto-temporal disconnection is directly associated with the positive symptoms of schizophrenia (Ford and Mathalon, 2005), we assume that normalization of fronto-temporal connectivity could be considered an experimental marker of an antipsychotic-like effect.

Animal studies on the role of 5-HT_1A_ receptors have shown that a1A had no effects on discriminative stimulus control by psilocybin in rats (Winter et al., 2007) but, in contrast, reversed the inhibitory effect of psilocin on locomotor activity, hole poking and time spent in the center (Halberstadt et al., 2011; Tyls et al., 2016). The activation of the 5-HT_1A_ receptor thus apparently represents an essential behavioral component of the effects of psilocin. An effect of a1A on EEG power and coherence that is similar to our observations was already shown in experiments with 5-methoxy-*N,N*-dimethyltryptamine (5-MeO-DMT), an indoleamine structurally similar to psilocin (Riga et al., 2016, 2018), which is known to be a psychedelic with a high affinity toward 5-HT_1A_ receptors. Our findings of EEG power increases in the 1–20 Hz frequency range after a1A alone are comparable with other studies (Marrosu et al., 1996) as well. On the other hand, an overall power increase in studies using 5-HT_1A_ agonists was also reported (Bogdanov and Bogdanov, 1994; Seifritz et al., 1996; McAllister-Williams and Massey, 2003). This discrepancy could be explained by differential involvement of pre/post-synaptic receptors and the differences between the effects of full/partial agonists (8-OH-DPAT, buspirone) and silent antagonists such as a1A used in our experiments. 5-HT_1A_ are inhibitory receptors located either pre-synaptically as somatodendritic autoreceptors in the raphe nuclei or post-synaptically in other brain regions (Hamon et al., 1990). The activation of presynaptic receptors results in an inhibition of serotonergic tone while the activity at postsynaptic receptors affects brain areas such as the hippocampus, lateral septum and various cortical areas (particularly the cingulate and entorhinal cortex) and they modulate the activity of glutamatergic and cholinergic neurons (Barnes and Sharp, 1999). While silent antagonists do not exert any particularly significant effects via presynaptic mechanisms, at the postsynaptic level they could, in fact, trigger inhibitory effects similar to the presynaptically-induced decrease in serotonergic tone. Psychedelics, as typical partial agonists, reduce serotonin release via presynaptic 5-HT_1A_ agonism (Riga et al., 2016) and, at the same time, inhibit the firing of cortical neurons (Araneda and Andrade, 1991), thus partially opposing the 5-HT_2A_ agonism. Nevertheless, since the postsynaptic 5-HT_1A_ and 5-HT_2A_ receptors are both expressed in pyramidal and GABAergic interneurons in the cortex (Santana et al., 2004) and show high cellular co-expression and functional interaction (Amargós-Bosch et al., 2004), there is no doubt of a complex interplay between them (Moreau et al., 2010).

Similarly to a1A, a2C does not block the psilocybin discriminative stimulus (Winter et al., 2007) and does not affect the psilocin-induced decrease in hole poking, time spent in the center, and PPI (Halberstadt et al., 2011). Regarding locomotor activity, the available findings are not consistent: while Halberstadt et al. (2011) observed no effect, in our previous study we have seen a normalization of the inhibitory effect (Tyls et al., 2016). The 5-HT_2C_ receptor also partially modulates rats’ 5-HT_2A_ receptor-induced head-twitch response (Vickers et al., 2001). Here we have shown a minor contribution of 5-HT_2C_-mediated mechanisms on overall EEG changes. As the 5-HT_2C_ receptor controls tonic dopamine release (Di Matteo et al., 1999; De Deurwaerdère and Di Giovanni, 2017) and can also elevate the extracellular levels of noradrenaline (Millan et al., 2005), its partial reversal potency might be explained at the network level by the engagement of other monoaminergic systems and, possibly, by the co-expression of 5-HT_2A/C_ receptors at the same GABAergic terminals (Nocjar et al., 2015). Separately, a2C leads to increased spectral power with delayed onset, as documented elsewhere (Kantor et al., 2005; Papp et al., 2020).

### 4.3. The effects of antipsychotics on EEG changes induced by psilocin

Haloperidol, a potent D_2_ receptor antagonist (Richelson and Souder, 2000), produces only minimal blockade of the subjective and behavioral effects of psilocybin in human volunteers (on the contrary it potentiates the anxiety induced by psilocybin) (Vollenweider et al., 1998a). Since the main mechanism of its action is dopaminergic, predictably, HAL had no impact on discriminative stimulus control of another serotonergic psychedelic LSD (Kuhn et al., 1978) and was only shown to attenuate the effects on PPI induced by DOI (Sipes and Geyer, 1994) but not by LSD (Ouagazzal et al., 2001). In a rat study, the administration of HAL significantly attenuated the influence of 5-MeO-DMT on low-frequency oscillations (up to 5 Hz) in PFC (Riga et al., 2016) but only partially affected the same effect induced by DOI (Celada et al., 2008). Surprisingly, our results demonstrate the partial ability of HAL to normalize psilocin-induced decrease in power and coherence similarly to other agents. Also, consistent with our results, a reduction in low and high frequencies as contrasted with an increase of the mid-frequencies was described for HAL alone in both humans (Czobor and Volavka, 1992) and rats (Sebban et al., 1999). Although psilocin has no affinity for dopamine D_2_ receptors, a PET study has shown that it alters dopamine transmission in the striatum (Vollenweider et al., 1999), which could account for the possible interactions and explain the partial efficacy of HAL.

Clozapine, as a potent 5-HT_2A/C_ and 5-HT_1A_ antagonist with further affinity for adrenergic, muscarinic and histamine receptors (Richelson and Souder, 2000), effectively abolishes discriminative stimulus effects of psychedelics (Schreiber et al., 1994); however, it has no effect on PPI deficits (Johansson et al., 1995). Indeed, we have seen the reversal of some changes induced by psilocin, but these were not equivalent to a simple summation of the effects of the individual antagonists. In our setting, CLO effected a robust increase in EEG power, particularly in the prefrontal cortex at 2–20 Hz, an effect possibly related to its α1 agonist activity (Sebban et al., 1999). Such increase in low-frequency oscillations as well as its epileptogenic potential is also a characteristic finding in patients treated with CLO (Centorrino et al., 2002). The robust effect within these frequency bands, which is in the opposite direction in contrast to psilocin, would obviously overcome any psilocin-induced changes in this frequency range. Most likely via the same mechanism and unlike HAL, CLO induced a full reversal of DOI-evoked reduction in this frequency range (Santana et al., 2004; Celada et al., 2008). Our study extends these findings, as we have seen a reversal of psilocin effects for the entire width of the EEG spectrum analyzed, including partial normalization of the power decreases within high beta and gamma oscillations. This effect, unique to CLO, can be explained by its interaction with muscarinic and histamine receptors–muscarinic antagonists have been shown to increase gamma oscillations in cats (Castro-Zaballa et al., 2019) and histaminergic modulation of these oscillations was described in the rat hippocampus (Fano et al., 2011). Changes in connectivity were influenced to some extent without further CLO-induced effects.

## 5. Conclusion

In our study, we investigated individual receptor contributions to psilocin-induced quantitative EEG changes. The effect of psilocin on EEG power and coherence, comparable to other psychedelics (Vejmola et al., 2021), was reversed to some extent by all agents used with respect to EEG power. Interestingly, the selective 5-HT_2*A*_ antagonist was the only agent effective in normalization of the disconnection induced by psilocin.

Our findings are in accordance with the early experiments evaluating the effect of psychedelics on EEG in animal and human subjects as well as with the recent neuroimaging studies with psilocybin. These data confirm the translational validity of the serotonergic model of acute psychosis in qEEG, which allows for further study of the role of brain networks in the neurobiology of human consciousness.

## Data availability statement

The original contributions presented in this study are included in the article/Supplementary material, further inquiries can be directed to the corresponding authors.

## Ethics statement

Ethical review and approval were not required for the animal study because all experiments respected the guidelines of the European Union (86/609/EU) and followed the instructions of the National Committee for the Care and Use of Laboratory Animals.

## Author contributions

FT, TP, ČV, and JH designed the research. FT, ČV, LK, and TP did the surgeries and EEG recordings. FT, ČV, VP, MBo, and TP preprocessed and analyzed the data. VK, VP, TN, and TP did the statistical analysis. FT, TP, and MBr wrote the initial draft of the manuscript. FT, ČV, VK, MK, ZB, MBr, JH, and TP contributed to the discussion of the results. All authors contributed to the article and approved the submitted version.

## Footnotes

1
https://github.com/vaclavapiorecka/BRAINBOX

2
http://www.3dbar.org/

## Supplementary material

Supplementary Figure 1 Impact of 5-HT antagonists and antipsychotics on qEEG induced by psilocin–relative differences to psilocin. The effects of treatments in three separate time epochs, as indicated. The abbreviations represent the following: SAL (control group, saline 2 ml/kg), PSI (psilocin, 100 mg/kg), a1A (5-HT_1A_ antagonist, 1 mg/kg), a2A (5-HT_2A_ antagonist, 0.5 mg/kg), a2C (5-HT_2C_ antagonist, 1 mg/kg), CLO (clozapine 5 mg/kg), HAL (haloperidol 0.1 mg/kg). **(A)** EEG topographic maps of absolute power spectra differences–all treatments vs. the psilocin group, drugs in rows, frequency bands in columns; only significant changes are indicated. The direction of change is indicated by colors–decreases in blue, increases in red. The level of significance is indicated by a three-level-scaled color spectrum (see legend). **(B)** EEG phase-lagged coherence topographic maps. All treatments are compared against the psilocin group, drugs in rows, frequency bands in columns. Only significant changes are indicated (*p* < 0.05). The direction of change is indicated by colors–decreases in blue, increases in red. The level of relative difference is indicated by a three-level-scaled line width. Corresponding legends **(A,B)** are at the bottom of the figure. Data were corrected for multiple comparisons via Bonferroni *post-hoc* test in case of time and treatment correction.
