# Supplementary figures and images for: Underlying pharmacological mechanisms of psilocin-induced broadband desynchronization and disconnection of EEG in rats
Source: Front Neurosci. 2023 Jun 22;17:1152578. doi: 10.3389/fnins.2023.1152578 (PMC10325866; doi:10.3389/fnins.2023.1152578)

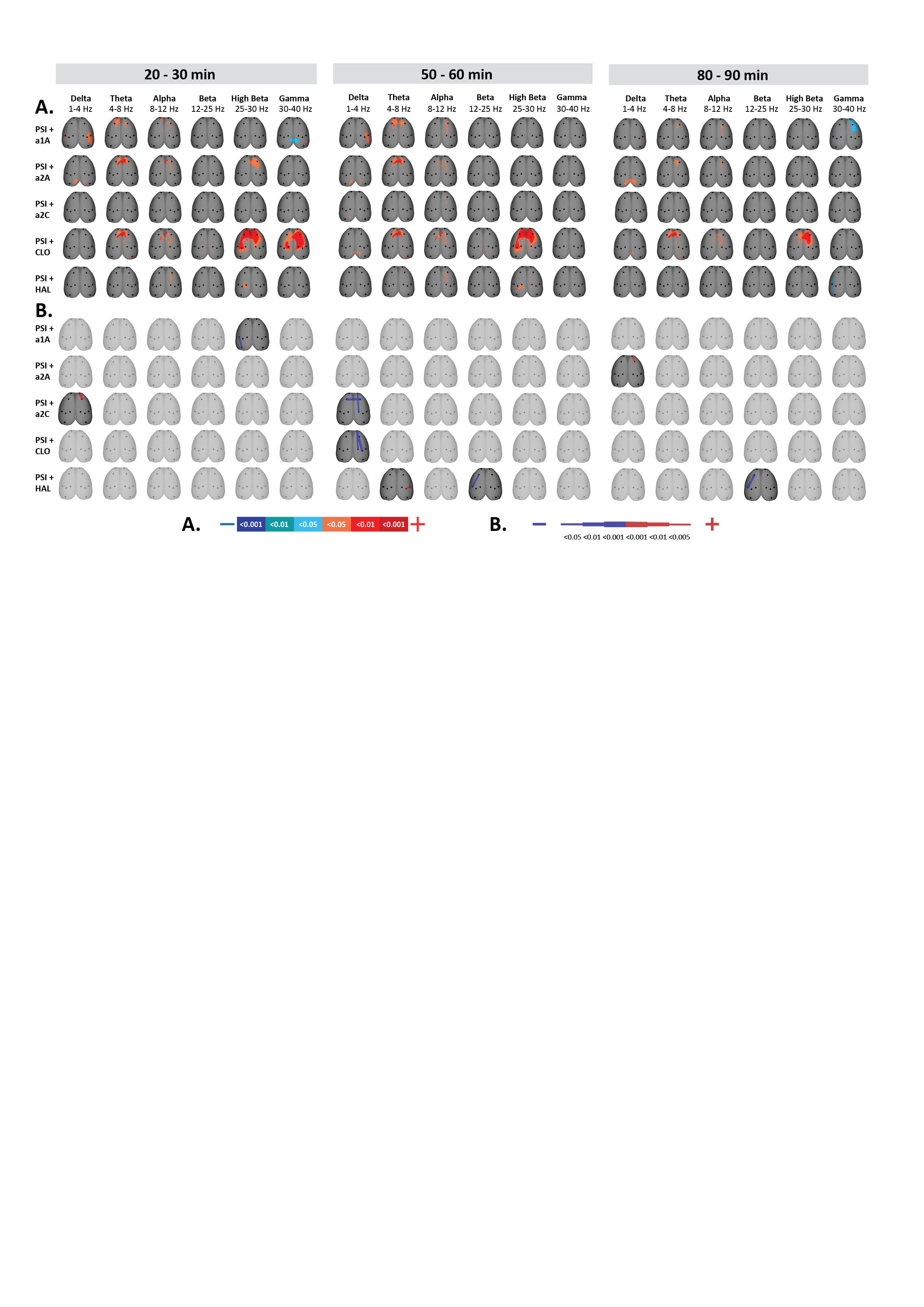

Supplement: Supplementary Figure 1 — Impact of 5-HT antagonists and antipsychotics on qEEG induced by psilocin–relative differences to psilocin. The effects of treatments in three separate time epochs, as indicated. The abbreviations represent the following: SAL (control group, saline 2 ml/kg), PSI (psilocin, 100 mg/kg), a1A (5-HT1A antagonist, 1 mg/kg), a2A (5-HT2A antagonist, 0.5 mg/kg), a2C (5-HT2C antagonist, 1 mg/kg), CLO (clozapine 5 mg/kg), HAL (haloperidol 0.1 mg/kg). (A) EEG topographic maps of absolute power spectra differences–all treatments vs. the psilocin group, drugs in rows, frequency bands in columns; only significant changes are indicated. The direction of change is indicated by colors–decreases in blue, increases in red. The level of significance is indicated by a three-level-scaled color spectrum (see legend). (B) EEG phase-lagged coherence topographic maps. All treatments are compared against the psilocin group, drugs in rows, frequency bands in columns. Only significant changes are indicated (p < 0.05). The direction of change is indicated by colors–decreases in blue, increases in red. The level of relative difference is indicated by a three-level-scaled line width. Corresponding legends (A,B) are at the bottom of the figure. Data were corrected for multiple comparisons via Bonferroni post-hoc test in case of time and treatment correction. [file Image_1.jpg]
